# An association between milk and slime increases biofilm production by bovine *Staphylococcus aureus*

**DOI:** 10.1186/s12917-015-0319-7

**Published:** 2015-01-16

**Authors:** Mary Hellen Fabres-Klein, Mário Junior Caizer Santos, Raphael Contelli Klein, Guilherme Nunes de Souza, Andrea de Oliveira Barros Ribon

**Affiliations:** Departamento de Bioquímica e Biologia Molecular, Laboratório de Biotecnologia Molecular, Universidade Federal de Viçosa, Viçosa, 36570-000 Minas Gerais Brazil; Embrapa Dairy Cattle, Juiz de Fora, 36038-330 Minas Gerais Brazil

**Keywords:** *Staphylococcus aureus*, Biofilm, Slime, Skim-milk medium, *agr* type

## Abstract

**Background:**

*Staphylococcus aureus* is associated with chronic mastitis in cattle, and disease manifestation is usually refractory to antibiotic therapy. Biofilm production is a key element of *S. aureus* pathogenesis and may contribute to the treatment failure that is consistently reported by veterinarians. Minas Gerais State is the largest milk-producing state in Brazil, and the characterization of bacterial isolates is an important aspect of disease control for dairy farmers. Here, we investigated the potential of *S. aureus* isolated from bovine mastitis to produce slime and biofilm in a skim-milk medium and classified the isolates according to their *agr* type.

**Results:**

Slime was detected using the Congo Red agar (CRA) test in 35.18% (19/54) of the strains; however, 87.04% (47/54) of the strains were considered biofilm-positive based on crystal violet staining. Compared to TSB supplemented with 0.25% glucose, skim milk significantly increased the production of biofilm, but this effect was only observed in slime-producing strains. The bacteria belonged to *agr* groups I (12/54), II (34/54), III (6/54), and IV (2/54), and bacteria in *agr* group III were found to be stronger biofilm producers than those in groups I and II. Again, milk had a significant influence only on slime-positive *agr* I and II isolates, revealing an association between milk and slime.

**Conclusions:**

The present study demonstrated that skim-milk medium and slime production are two factors that together influence biofilm formation by bovine strains of *S. aureus*. A predominance of bacteria belonging to *agr* group II was observed, and bacteria from *agr* group III showed the highest proportion of biofilm producers. The majority of bacteria characterized in this study formed biofilm in milk, which suggests that biofilm formation has an important role in the virulence of *S. aureus* isolated from bovine intramammary infections.

## Background

*Staphylococcus aureus* is a pathogen that frequently causes mastitis in bovine herds worldwide. In Brazil, which is considered by the Food and Agriculture Organization (FAO) as the fourth largest milk producer in the world [1], *S. aureus* infections are also a major concern with respect to the welfare of dairy cattle. The presence of this pathogen in a dairy herd was first reported in Brazil in 1978 by Muller et al. [2]; since then, the pathogen has been found in herds distributed throughout the country [3,4]. In Minas Gerais State, over 220,000 farms are engaged in milk production, indicating the supremacy of this region in the milk production sector [5]. However, subclinical mastitis accounts for a high percentage of the disease manifestations in Minas Gerais [6], and epidemiological studies have shown that the prevalence of *S. aureus* can reach nearly 50%.

Veterinarians describe intramammary infections caused by *S. aureus* as a subclinical manifestation that usually evolves into a chronic state [7]. One possible reason for the persistence of the pathogen in the udder is the formation of biofilms, which are bacterial communities attached to surfaces that are embedded in matrices mainly composed of polysaccharides [8]. The correlation between biofilms and the persistence of *S. aureus* of bovine origin has been previously described [9]. However, Simojoki et al. [10] evaluated nearly 200 coagulase-negative staphylococci associated with bovine mastitis and found that neither biofilm nor slime production are correlated with persistent infection. Another complaint by veterinarians is the low efficacy of antimicrobial treatments for infections caused by *S. aureus* [7], although it has been reported that inhibiting bacterial biofilm production reduces bacterial resistance in *in vivo* assays [11]. Recently, Beenken et al. [12] demonstrated that reduced biofilm production by *S. aureus* in a murine model was correlated with increased susceptibility to daptomycin.

The production of biofilm depends on the ability of bacteria to attach to abiotic/biotic surfaces, proliferate, and produce an extracellular matrix, which is mainly formed by polysaccharide intercellular adhesion (PIA) in *S. aureus* [13]. PIA is the major component of the extra-polysaccharide matrix, also known as slime, and is encoded by the *ica* operon (i*caABCD*). Deletion of this operon results in impairment of biofilm formation and the production of PIA *in vitro* [14]. An indirect relationship between the *ica* locus and the accessory gene regulator (*agr*) locus, a quorum-sensing system that regulates the expression of several virulence traits in *S. aureus,* has been found. Some reports have shown that low *agr* activity is needed to support biofilm development but that the dispersion of bacterial cells relies on the secretion of proteases, which is stimulated by *agr* activation [15,16].

The *agr* system can be used to divide *S. aureus* into four groups. Buzzola et al. [17] found a high prevalence (88%) of *agr* group I in bovine mastitis isolates from Argentina, and this group was also prevalent (69%) in the studies of Gilot and van Leeuwen [18], which describe the analysis of isolates from different countries. In contrast, 81% of the field strains collected by Melchior et al. [19] in the Netherlands belonged to *agr* type II, while 9% belonged to *agr* I. In this last report, the authors showed that *agr* II strains produce more biofilm in milk serum than *agr* I strains and suggested clinical implications for these observations, such as the adaptation of *agr* II to the extracellular niche.

In this study, we evaluated the ability of Brazilian isolates to produce slime and biofilm and classified them according to their *agr* type. Our results show that milk and slime production are two factors that have a positive effect on biofilm production by bovine isolates of *S. aureus*, regardless of their *agr* type.

## Results

**Figure 1:**
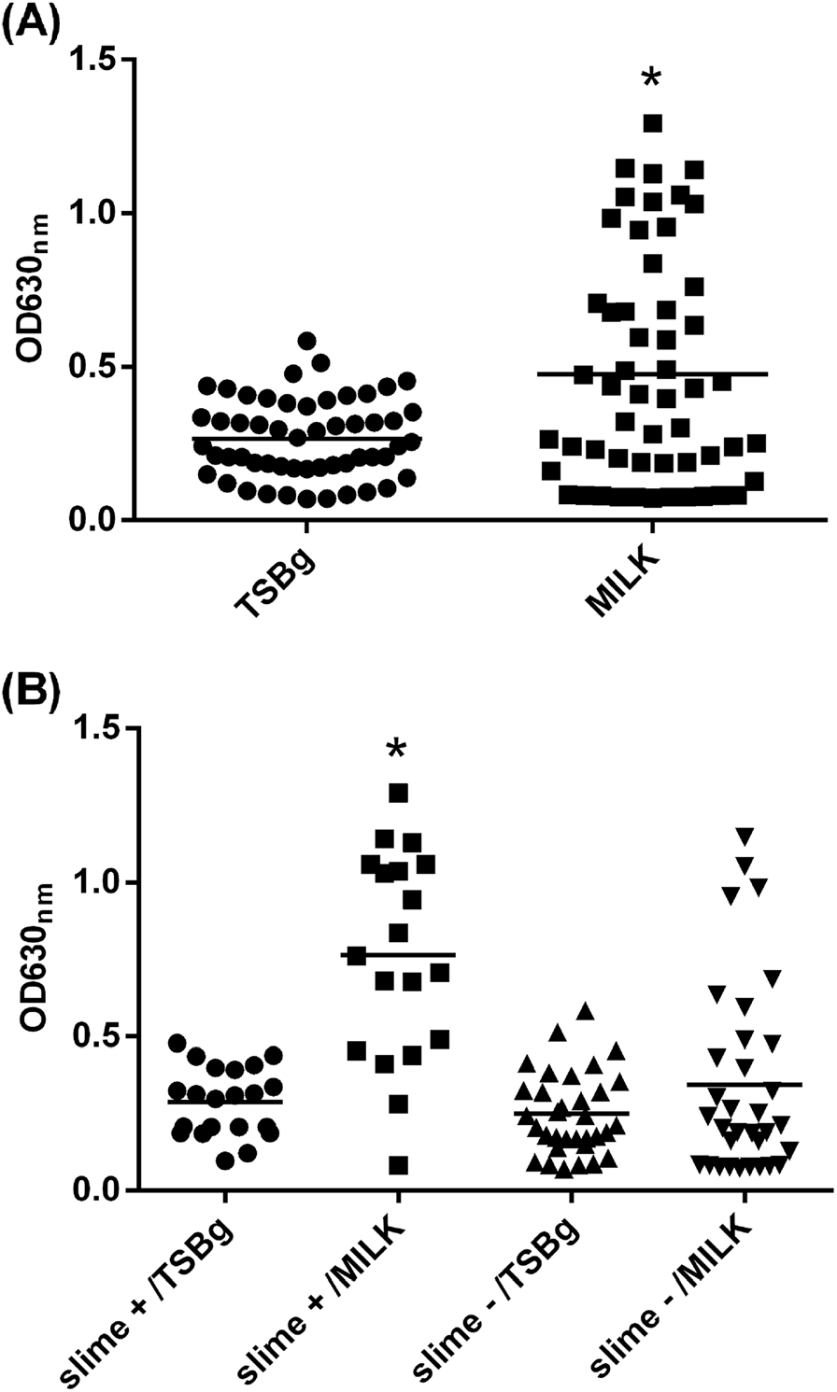
**Skim milk and slime increase the production of biofilm by bovine** ***Staphylococcus aureus*** **strains.** Bacteria were grown in polystyrene microtiter plates in TSB supplemented with 0.25% glucose or skim-milk medium, and biofilm production was visualized by crystal violet staining. Isolates cultivated in skim-milk medium **(A)** and isolates with a slime-positive phenotype grown in skim-milk medium **(B)** showed increases in biofilm production. The horizontal bar represents the average biofilm production. The experiments were performed in triplicate. * *P* < 0.001.

Fifty-four bovine isolates of *S. aureus* were screened for their ability to produce slime and biofilm in two different media. *Streptococcus agalactiae* was used as a negative control for slime and biofilm production due to the phenotype of its colonies on Congo Red agar (CRA; red-smooth colonies) and because it showed the same optical density (OD_630_) value as the medium without bacteria in microplate assays. Nineteen (35.18%) and thirty-five (64.82%) isolates were classified as slime producers and non-slime producers, respectively, according to the phenotype of the colonies on CRA. The majority of the isolates (47/54) produced biofilm in TSBg (Trypticase soy broth containing 0.25% glucose), a medium that is known to stimulate biofilm production [20]. Skim milk also promoted the production of biofilm by 77.7% (42/54) of the isolates. However, the average production of biofilm by the 54 isolates in TSBg was lower when compared with their average biofilm production in skim-milk medium (*P* < 0.001) (Figure 1A). The difference was also significant (*P* < 0.001) when skim milk was used as the growth medium for the slime-producing strains (Figure 1B). Conversely, milk did not have a positive effect on the non-slime producers.

**Figure 2:**
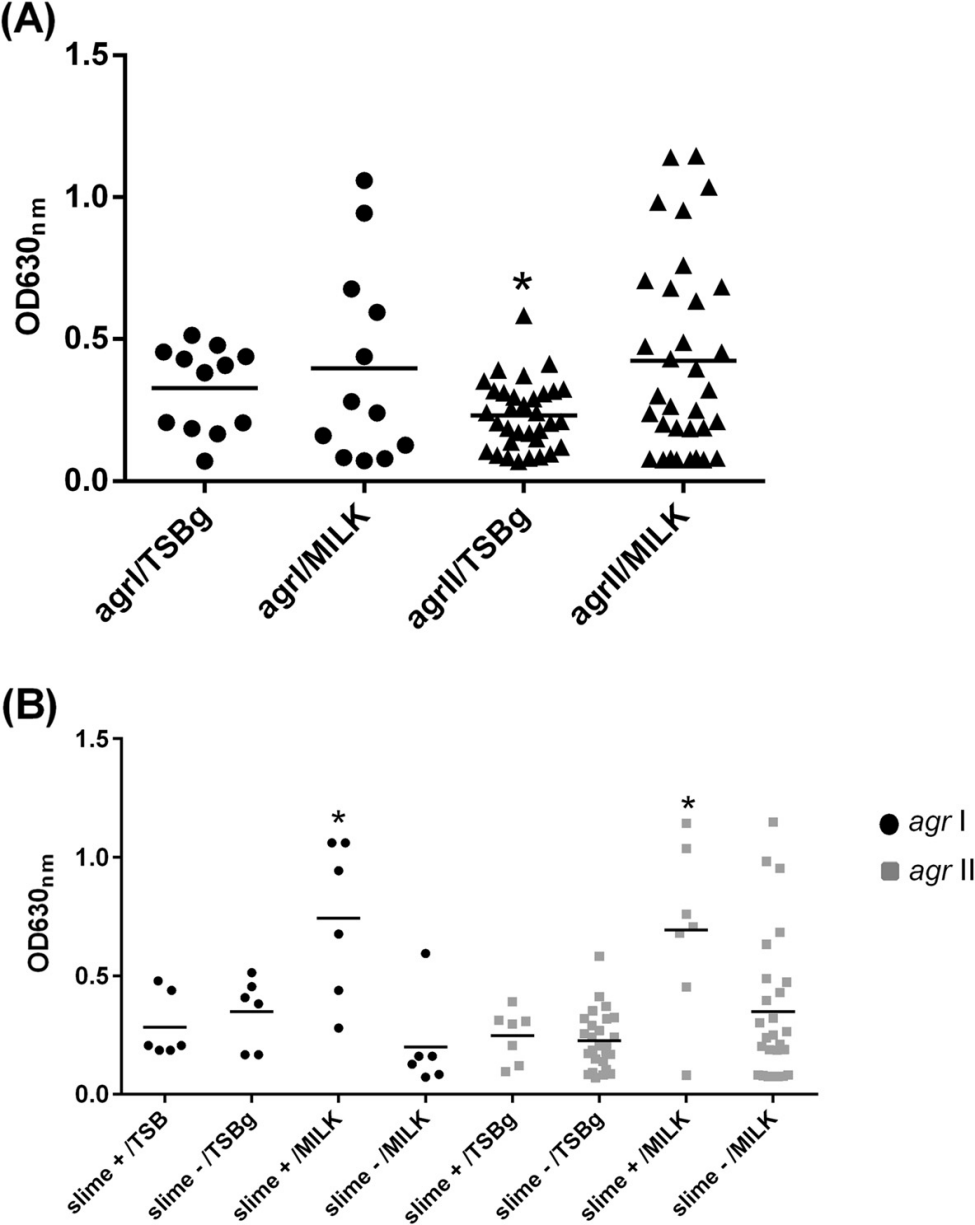
**Biofilm formation by** ***agr*** **type I and II** ***Staphylococcus aureus*** **strains.** Bacteria grown in skim-milk medium **(A)** and slime-positive strains cultivated in skim-milk medium **(B)** showed increases in biofilm production. The crystal violet staining method was used to detect biofilm production in polystyrene microplates. The experiments were performed in triplicate. * *P* < 0.05.

According to the *agr* type, the strains were classified as follows: *agr* I (12/54), *agr* II (34/54), *agr* III (7/54), and *agr* IV (2/54). Biofilm production by *agr* group I did not differ significantly regardless of the medium used (TSBg or milk) (*P* = 0.557) (Figure 2A). Nevertheless, skim milk promoted biofilm production by *agr* group II.

An effect of the culture medium on the amount of biofilm formed by the slime-positive *agr* I strains was observed (*P* = 0.028), suggesting that slime also exerted an influence on the production of biofilm (Figure 2B). The same result was not observed in strains that were classified as non-slime producers. The results for *agr* group II were similar; again, only the slime-positive group showed an increase in biofilm production in milk (p = 0.015). No significant difference between the slime-positive or slime-negative strains was detected in TSBg (p = 0.699). There was a significant difference in biofilm production between slime-positives from *agr* I and *agr* II groups when grown in skim-milk medium (*agr* I = 0.6, *agr* II = 0.69) compared to growth in TSBg (*agr* I = 0.26, *agr* II = 0.24).

**Figure 3:**
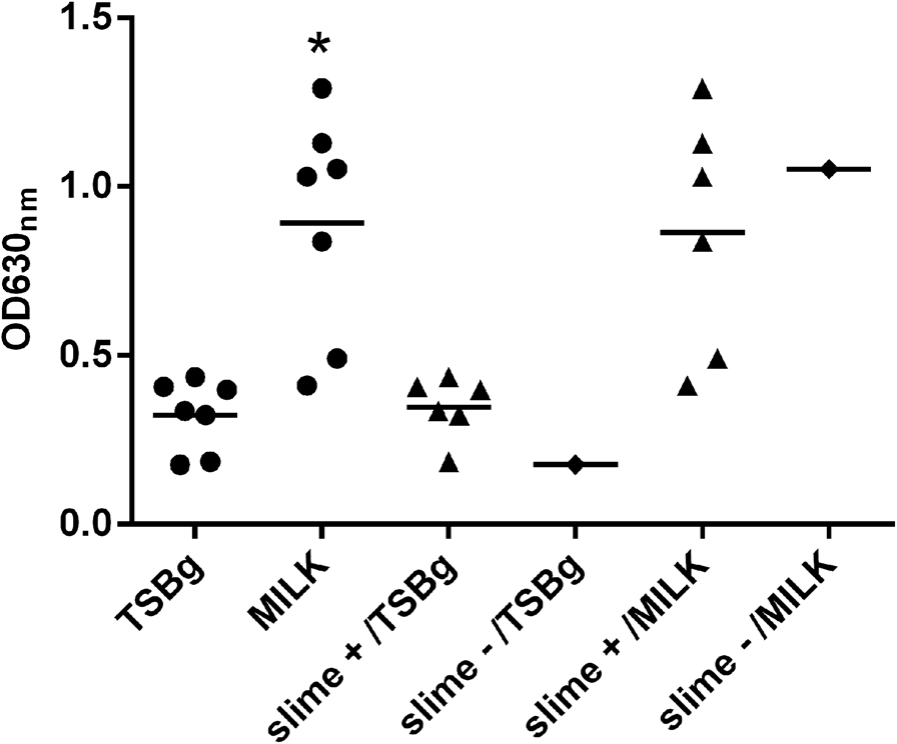
**Biofilm production by** ***agr*** **group III** ***Staphylococcus aureus*** **strains is significantly increased in skim-milk medium.** Bacteria were grown in polystyrene microtiter plates in TSB supplemented with 0.25% glucose (TSBg) or skim-milk medium (Milk). The crystal violet staining method was used to detect biofilm production in the polystyrene microplates. The experiments were performed in triplicate. * *P* < 0.05.

Despite the small number of *agr* III isolates, milk had a positive effect (*P* < 0.05) on biofilm production (Figure 3) regardless of slime production. It should be noted that the average biofilm production of *agr* III (0.89) was higher than that of the other groups (0.6 and 0.69) in milk. All isolates (54/54) tested positive for the presence of the *icaAD* genes (data not shown).

## Discussion

In this study, the production of biofilm and slime by *S. aureus* isolated from mastitic cows was investigated. Although other authors have used biofilm and slime synonymously [21,22], slime is in fact a component of biofilm. “Slime formation” was once the term used for biofilm formation [23]; however, if we revisit the review published by Hall-Stoodley et al. [24], slime is defined as the extracellular polymeric substance, also known as EPS, that is mainly formed by PIA in *S. epidermidis* and *S. aureus,* although DNA and proteins can also be found in this material. Biofilm was defined by Costerton et al. [25] as a population of cells that is attached to a surface and enclosed by a matrix. In contrast, slime is the protective matrix that surrounds this population and has a fundamental role in the biofilm structure, as it maintains adhesion between the cells and acts as a protective barrier against the host immune system and biocides [24].

Among the *S. aureus* isolates tested in the present study, a minority (19/54) were found to be slime-positive, even though the majority (47/54) were considered to be biofilm producer. Only two slime-positive isolates (3885 and 688) did not produce biofilm in TSBg, yet biofilm was observed when they were grown in skim milk. This low correlation between biofilm and slime may be due to limitations of the CRA method, a qualitative test used to categorize bacterial strains as slime producers or non-slime producers based on the appearance of the colonies. A positive result is indicated by black colonies. The black color supposedly manifests due to a greater association between the thick layer of exopolysaccharides and the Congo Red stain; this association is decreased in slime-negative strains, resulting in a lighter color (red- or pink-Bordeaux). It is known that the thickness of the polysaccharide layer that surrounds the bacterial cell wall differs among *S. aureus* strains [26,27]. Thus, it is plausible that the CRA test lacks the appropriate sensitivity needed to discriminate strains that form thinner extracellular layers from those that do not produce the layer, instead placing both phenotypes in the same category (slime-negative). If we consider that a thinner layer still allows cell adhesion and promotes biofilm formation, the results from the CRA test would be similar those obtained in the crystal violet staining assay (87.04%).

The slime-producing abilities of bovine isolates of *S. aureus* have been reported to range from 11.42% to 91.42% [22,28], and differences in the criteria used for the interpretation of the CRA test (color, morphology or both) could explain this discrepancy [28]. A low correlation between the CRA test and the crystal violet assay was found by some authors, who attributed these findings to differences in the culture conditions used [29,30].

Biofilm production was increased in a skim-milk medium. Lactose and milk whey also contribute to capsule polysaccharide and biofilm formation in *S. aureus* [19,31]. Recently, Varhimo et al. [32] reported that milk components stimulated biofilm formation in *Streptococcus uberis*. When added at a low concentration into TSB medium, milk or lactose was also found to upregulate *ica* operon genes in two strains of *S. aureus* associated with bovine mastitis [33]; in one strain, milk also promoted an increase in the transcription of surface proteins such as Bap, the biofilm-associated protein. These studies suggest that bovine isolates of *S. aureus* adapt to the milieu found in the udder, with milk influencing biofilm production and hence promoting bacterial survival.

Milk had a positive effect on the *agr* group II and III strains but not on the *agr* I strains. It was previously shown that strains belonging to *agr* group I have an increased ability to invade and persist in MAC-T cells [17,34]; unlike other *agr* groups that might be more adapted for survival outside mammary gland cells, biofilm production might not be an important factor for the persistence of *agr* I bacteria in the udder. Strong biofilm producers were observed among other strains growing in extracellular niches, such as those isolated from catheter-associated infections, and found to belong to *agr* genotype II [15]. In our study, the few isolates from *agr* group III were considered to be strong biofilm producers in milk. However, this analysis should be extended to more isolates to confirm this pattern. Indeed, *agr* group III is always less prevalent in *S. aureus* isolated from bovine mastitis compared to *agr* groups I and II [17,19,18], and this may explain why *agr* group III is less studied. Similar to *agr* II, *agr* III isolates are less likely to be internalized by MAC-T cells [17]. This feature, combined with the high biofilm production observed in the present study, can suggest a better adaptation of these isolates to the extracellular milieu.

Biofilm formation is a protective mechanism used by bacteria to avoid antimicrobials, and this could contribute to mastitis treatment failure. Field isolates of *S. aureus* found to be susceptible to several antibiotics based on CLSI testing methods were considered highly resistant when grown in biofilms [35]. According to Raza et al. [36], the EPS secreted by the bacteria acts as a barrier that may play a role in this resistance, preventing the adsorption and penetration of antimicrobials. Alternatively, the EPS matrix could neutralize or bind these compounds, promoting their dilution to subinhibitory concentrations before they reach the cells [24]. Biofilms are composed of dormant and active cell subpopulations, and this difference in bacterial physiology can also influence the efficacy of antibiotics [37] and hence the outcome of mastitis therapy.

An interesting observation was the significant increase in biofilm formation by slime-producing bacteria in skim-milk medium, indicating a potential association between slime and milk. If we consider the invasive potential of *agr* I isolates [17], the presence of slime and milk could promote a change in the bacterial lifestyle that increases the chances of survival in the extracellular medium. Slime-producing strains of *agr* II also showed improved biofilm formation following growth in skim-milk medium. As another role of the EPS matrix is to increase adherence to the cell surface [38], this could stimulate the formation of biofilm by bovine isolates and, consequently, bacterial resistance to antimicrobials.

In this study, we showed that 87.04% (47/54) of the bovine isolates that were analyzed produced biofilm, which was well above the values that were found in herds from the USA (68.57%) [29], India (29.41%) [22], Portugal (37.5%) [30], and Poland (57.6%) [39]. However, unlike the study in Poland [39], which analyzed a population representing different genotypes with diverse abilities to form biofilms, the genetic background of the strains was not assessed in our study, which may have influenced the outcome. The ability of some genotypes to produce more biofilm than others has been demonstrated elsewhere [40]. Our results showed that all of the isolates carried the *icaA* and *icaD* genes, which was also reported for the *S. aureus* isolates from American herds [29]. Thus, the presence of these two genes in our isolates and in the American isolates could explain why our values were similar to those in the American study. In contrast, only 35.29% (36/102) of the strains harbored both genes in the study carried out in India [22]. Because the *ica* operon is responsible for the production of PIA, it is expected that *ica*-positive isolates are more likely to produce biofilm. The expression of these genes should be assessed in future studies to evaluate their actual contribution to biofilm production. Coexpression of the *icaA* and *icaD* genes is related to high activity of *N*-acetylglucosaminyltransferase, which is involved in PIA biosynthesis in *Staphylococcus epidermidis* [41]. In coagulase-negative staphylococci, there was concordance between the expression of *icaAD* and biofilm production [42].

## Conclusions

The present study demonstrated that slime-producing strains can produce more biofilm when grown in a skim-milk medium, suggesting an association between milk and slime. A predominance of bacteria belonging to *agr* group II was observed, and bacteria from *agr* group III were the best biofilm producers. All isolates were *icaAD-*positive, and most of them formed biofilm in milk. Considering the large number of biofilm-producing bacteria found in this work, it is suggested that biofilm production represents a challenge in the control of bovine mastitis in Minas Gerais.

## Methods

### Bacterial isolates

The 54 *S. aureus* isolates used in this study were kindly provided by Embrapa Dairy Cattle, Juiz de Fora, Minas Gerais. They were collected between 1996 and 2011 from cows with subclinical mastitis belonging to dairy herds located in Minas Gerais State. *S. aureus* was identified by microbiological methods and biochemical tests [43]. The reference strains *Staphylococcus epidermidis* NRS101, *S. aureus* NRS133 and *S. aureus* NRS155 were obtained from the Network on Antimicrobial Resistance in *Staphylococcus aureus* (NARSA) and were used as positive controls in the biofilm assays (NRS101) or *agr* typing (NRS133 and NRS155). *Streptococcus agalactiae*, which was isolated from a mastitic cow, served as the negative control. The bacteria were grown in brain heart infusion broth (BHI, HiMedia, Mumbai, India) or Trypticase soy broth (TSB; HiMedia) at 37°C with agitation. Skim milk powder (HiMedia) was also used as a growth medium in the biofilm assay. All isolates used in this study were stored at -70°C in BHI containing 40% glycerol.

### Slime production on Congo Red agar

Polysaccharide-producing *S. aureus* strains were determined by cultivation on agar plates containing Congo Red (Sigma-Aldrich, Oakville, Ontario, Canada) [22]. Initially, the bacteria were inoculated in BHI broth and incubated at 37°C for 16 h with agitation. The cultures were then streaked onto CRA plates and incubated at 37°C for 24 h and subsequently kept at room temperature for 48 h and 72 h. Only isolates growing as black colonies with a dry and crystalline consistency were considered to be slime producers. *S. epidermidis* NRS101 and *Streptococcus agalactiae* were used as positive and negative controls, respectively. The assay was repeated three times for each isolate.

### Biofilm production assay

Biofilm formation was assessed in 96-well polystyrene tissue culture microplates as previously described [29] with modifications. The bacterial isolates were inoculated into TSB and incubated at 37°C for 16 h, and the optical density (OD_600nm_) was adjusted to 0.1. Subsequently, the inoculum was diluted 1:40 in TSB containing 0.25% glucose (TSBg) or skim-milk medium in a final volume of 200 μL per well, and the microplate was incubated at 37°C for 24 h without agitation. The medium was then discarded, and the wells of each plate were gently washed three times with 200 μL of sterile PBS (pH 7.4), dried at 45°C for 20 min and then stained with 50 μL of 1% crystal violet for 15 minutes. Each well was washed three times with 200 μL of sterile distilled water, followed by drying at 45°C for 20 min; 200 μL of 100% ethanol was then added. A 150-μL aliquot was removed from each well and transferred to a new microplate, and the absorbance at 630 nm was measured using a microplate reader (VersaMax Molecular Devices, Sunnyvale, California, USA). Wells filled with TSBg or skim-milk medium were used as blanks to correct for background staining by subtracting the value of the blank from each experimental value. The bacterial isolates were classified as biofilm-positive if their average OD values were higher than the average OD value of the negative control (*Streptococcus agalactiae*) (OD_630nm_ values < 0.1). *S. epidermidis* NRS101 was used as the positive control. Each isolate was tested in triplicate, and the assay was repeated three times.

### Polymerase chain reaction

Genomic DNA from the bacterial isolates was extracted according to Pospiech & Neumann [44]. The DNA quality and quantity were analyzed by gel electrophoresis. A total of 150 ng μL^-1^ of DNA was used in amplification reactions with specific primers (Table 1). The reactions contained 1X reaction buffer, 1.5 mM MgCl_2_, 0.2 mM dNTPs, 1U GoTaq DNA polymerase (Promega, Madison, Wisconsin, USA), 1 μM each primer and Milli-Q water to increase the reaction volume to 20 μL. The cycling conditions were either the same as those suggested by Ciftci et al. [45] to amplify the *icaA* and *ica*D genes or the same as those suggested by Campbell et al. [46] to amplify different *agr* groups. *S. aureus* NRS133 and NRS155 were used as positive controls for *agr*-types I and II, respectively. The amplicons were resolved by 1.5% agarose gel electrophoresis on gels containing 0.5 μg mL^-1^ ethidium bromide. Images were registered using the L-PIX Imaging system (Loccus Biotecnologia, São Paulo, São Paulo, Brazil).

**Table 1:** **Primers used in this study**

**Gene**	**Primer sequence**	**Concentration (μM)**	**Product size (bp)**	**Tm (°C)**	**Reference**
*icaA*	F- CCTAACTAACGAAAGGTAG	1.0	1315	49	[45]
	R- AAGATATAGCGATAAGTGC				
*icaD*	F- AAACGTAAGAGAGGTGG	1.0	381	49	[45]
	R- GGCAATATGATCAAGATA				
*agr*-type I	F-ATCGCAGCTTATAGTACTTGT	1.0	578	53	[46]
	R-CTTGATTACGTTTATATTTCATC				
*agr*-type II	F-AACGCTTGCAGCAGTTTATTT	1.0	814	57	[46]
	R-CGACATTATAAGTATTACAACA				
*agr*-type III	F-TATATAAATTGTGATTTTTTATTG	1.0	893	53	[46]
	R-TTCTTTAAGAGTAAATTGAGAA				
*agr*-type IV	F-GTTGCTTCTTATAGTACATGTT	1.0	757	53	[46]
	R-CTTAAAAATATAGTGATTCCAATA				

### Statistical analysis

Data analyses were performed using the statistical programs SAS/STAT® [47] and SPSS [48]. Descriptive statistics (the arithmetic mean, standard deviation and standard error) were used to assess biofilm production in different media (TSB or skim milk) and by different *agr* groups (I, II, or III). To compare the mean biofilm production values according to the medium and *agr* group, a two-way factorial ANOVA was used; the least significant difference (LSD) test was employed for comparison of the means. The two-way factorial model used for the slime-positive isolates and biofilm producers in TSBg and skim milk was as follows: Y_ij_ = m + MED_i_ + AGR_J_ + MEDxAGR_k_ + e_ijk_, where Y_ij_ = observed values (slime), m = constant associated with each observation, MED_i_ = the fixed effect of the medium i (milk = 0 and TSB = 1), AGR_J_ = the fixed effect of the *agr* group (*agr* II = 0 and *agr* I and III = 1), MED x AGR_k_ = the interaction of the medium and the *agr* group, and e_ijk_ = random error. The OD values for the production of biofilm in TSB or skim milk were categorized as negative (OD ≤ 0.1) or positive (OD > 0.1). We used Fisher's exact test and the Kappa index to evaluate the association and level of agreement between the positive and negative results for biofilm production and the positive and negative results for slime production (SPSS, 1998). The variation in slime production was assessed by generalized linear models. The values for slime production were zero (0) for a negative result and one (1) for a positive result. Slime production by each isolate was measured. The biofilm production in the respective growth medium was categorized as positive or negative, and isolates were categorized as “*agr* I and III” or “*agr* II”.

## Abbreviations

CRA: Congo Red agar
FAO: Food and agriculture organization
PIA: Polysaccharide intracellular adhesion
EPS: Extracellular polymeric substance
OD: Optical density
BHI: Brain heart infusion
TSB: Trypticase soy broth
TSBg: Trypticase soy broth containing 0.25% glucose
NARSA: Network on antimicrobial resistance in *Staphylococcus aureus*
